# Supplementary material for: The interactome of intact mitochondria by cross-linking mass spectrometry provides evidence for coexisting respiratory supercomplexes
Source: Mol Cell Proteomics. 2017 Dec 8;17(2):216–32. doi: 10.1074/mcp.RA117.000470 (PMC5795388; doi:10.1074/mcp.RA117.000470)
Supplement: Supplemental Data [file supp_RA117.000470_133922_0_supp_23978_fzffwf.zip › spectra_annotation/mito_spectra_annotation/355-1-4-1-15-1.pdf]

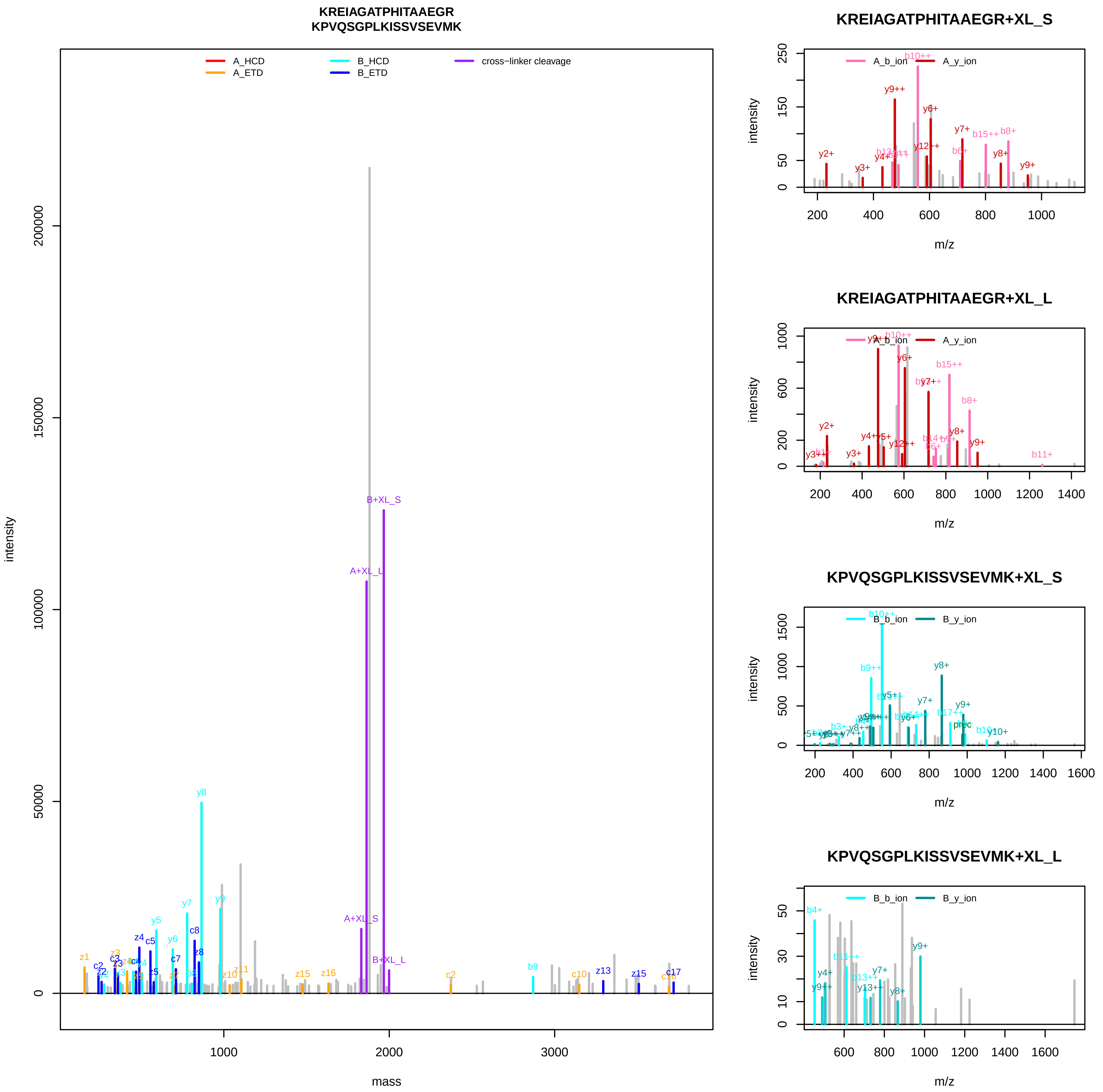
KREIAGATPHITAAEGR
KPVQSGPLKISSVSEVMK
A_HCD
A_ETD
B_HCD
B_ETD
cross−linker cleavage
intensity
mass
0
50000
100000
150000
200000
1000
2000
3000
B+XL_S
A+XL_L
A+XL_S
B+XL_L
y8
y7
y9
y5
y6
c8
z4
c5
c7
z8
z1
z3
c3
c2
z2
c4
z5
b9
c2
c10
z13
z15
c17
c16
z15
z16
z10
z11
KREIAGATPHITAAEGR+XL_S
A_b_ion
A_y_ion
m/z
b10++
y9++
y6+
y7+
b15++
b8+
y12++
y2+
y3+
y4+
b6+
y8+
y9+
KREIAGATPHITAAEGR+XL_L
b15++
b8+
y2+
y3++
y3+
y4+
y5+
y12++
y7++
y8+
y9+
b11+
KPVQSGPLKISSVSEVMK+XL_S
B_b_ion
B_y_ion
b10++
b9++
y8+
y7+
y9+
y5+
b17++
y6+
y10+
prec
KPVQSGPLKISSVSEVMK+XL_L
b4+
b11++
y9+
y4+
y9++
y7+
b13++
y13++
y8+
